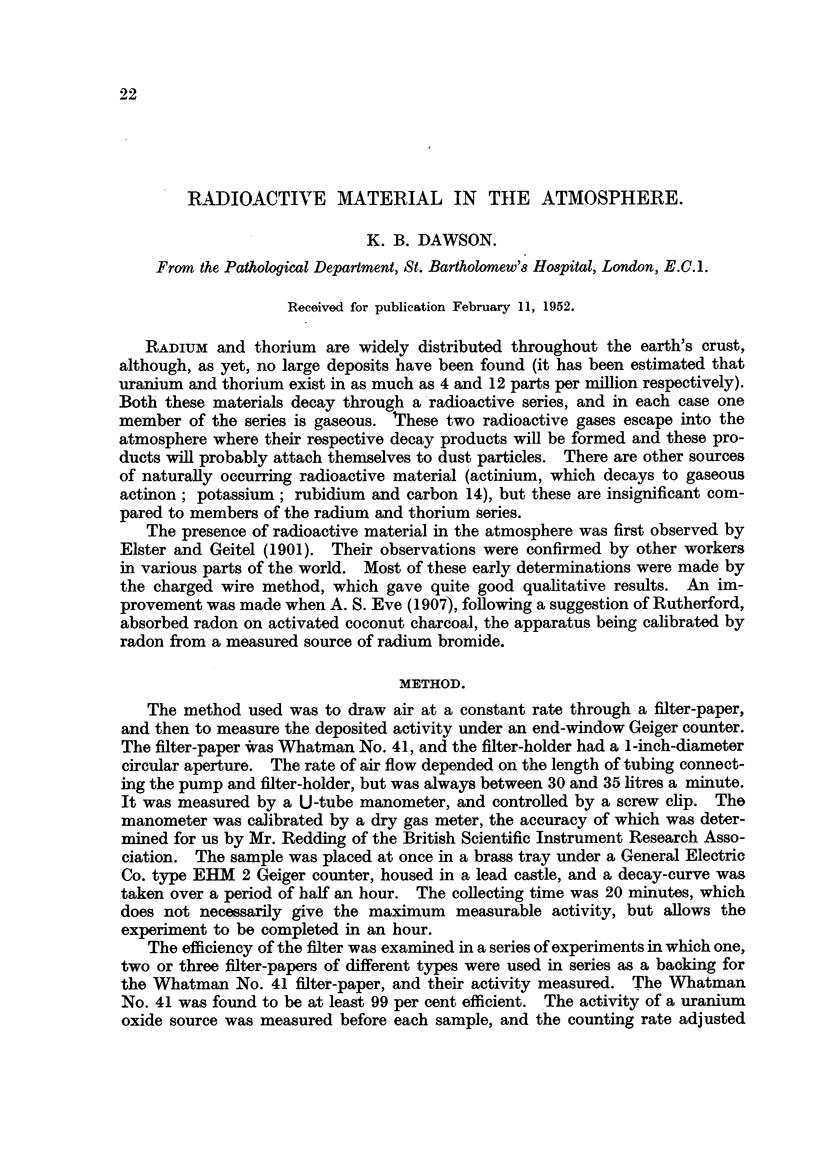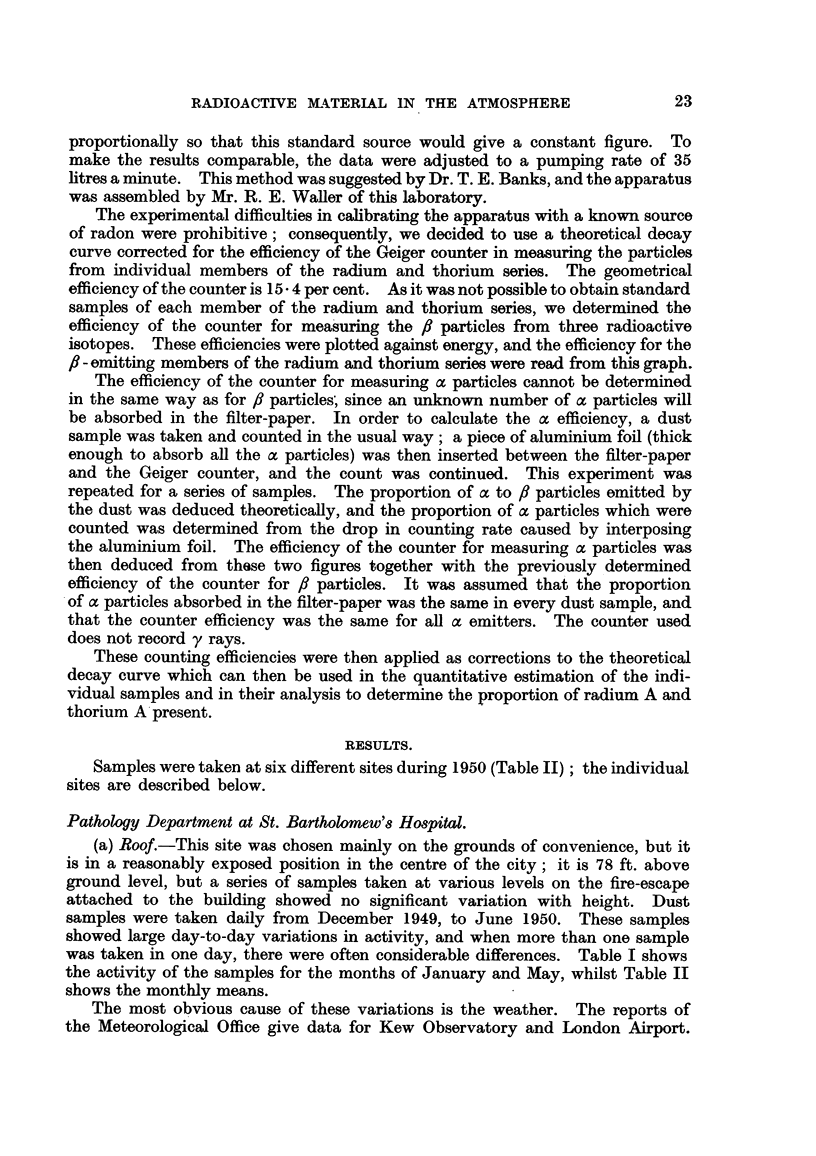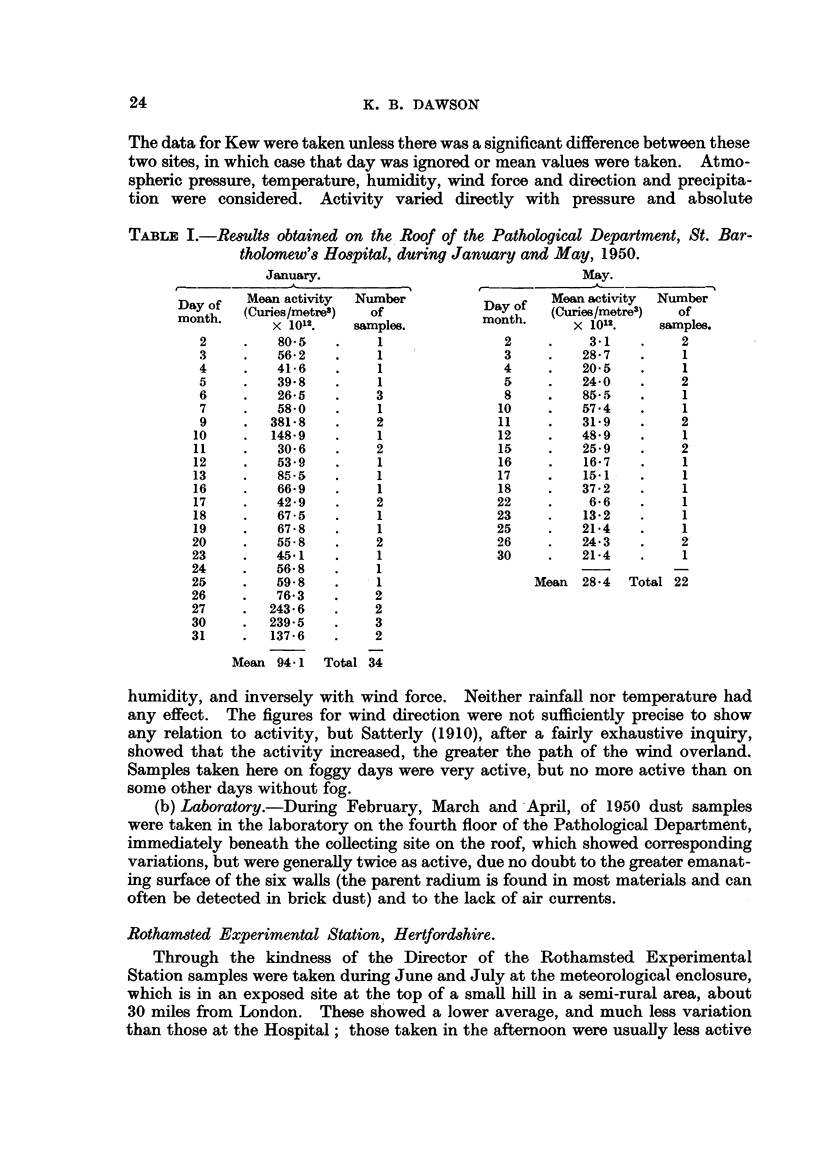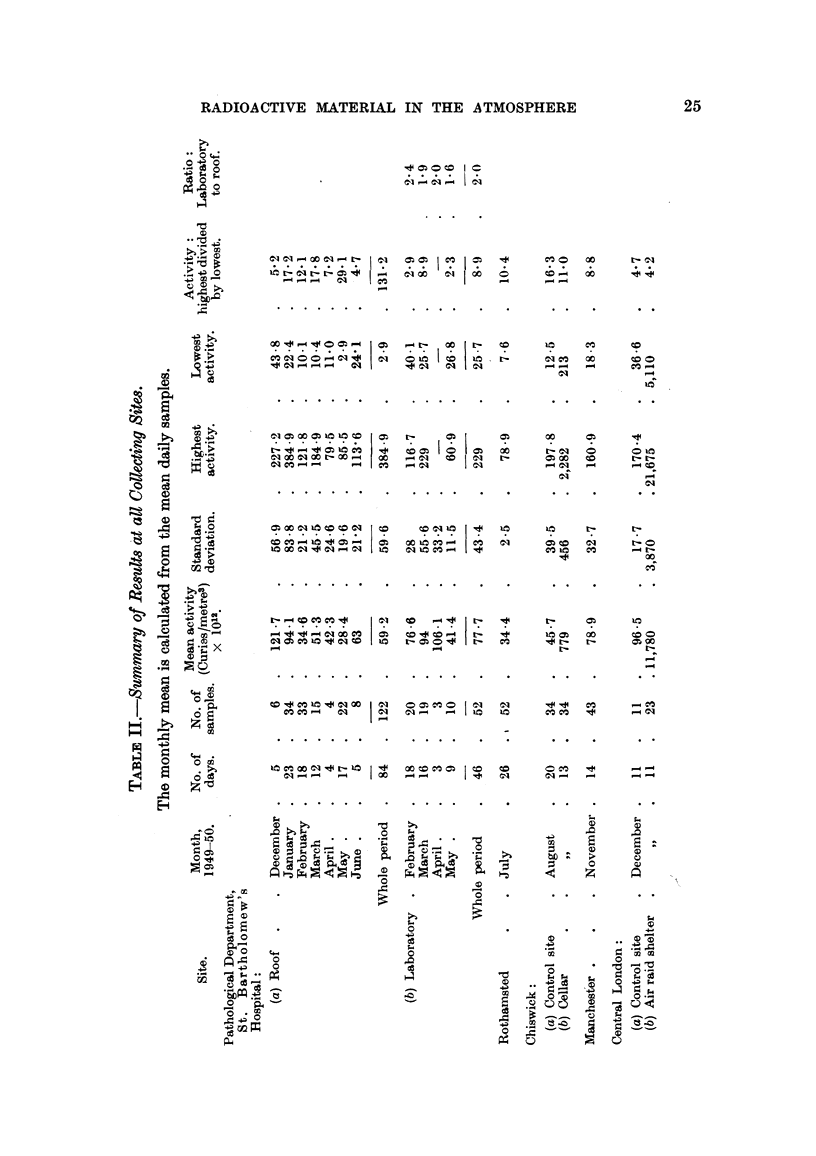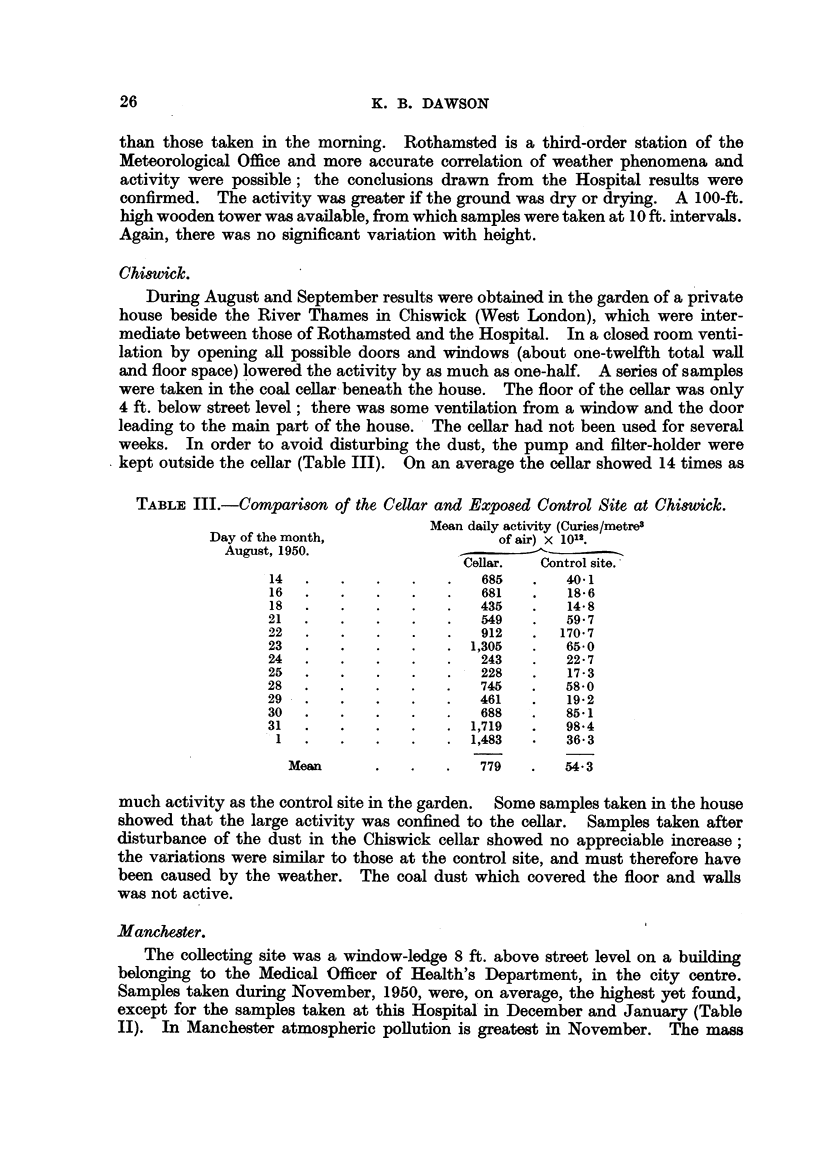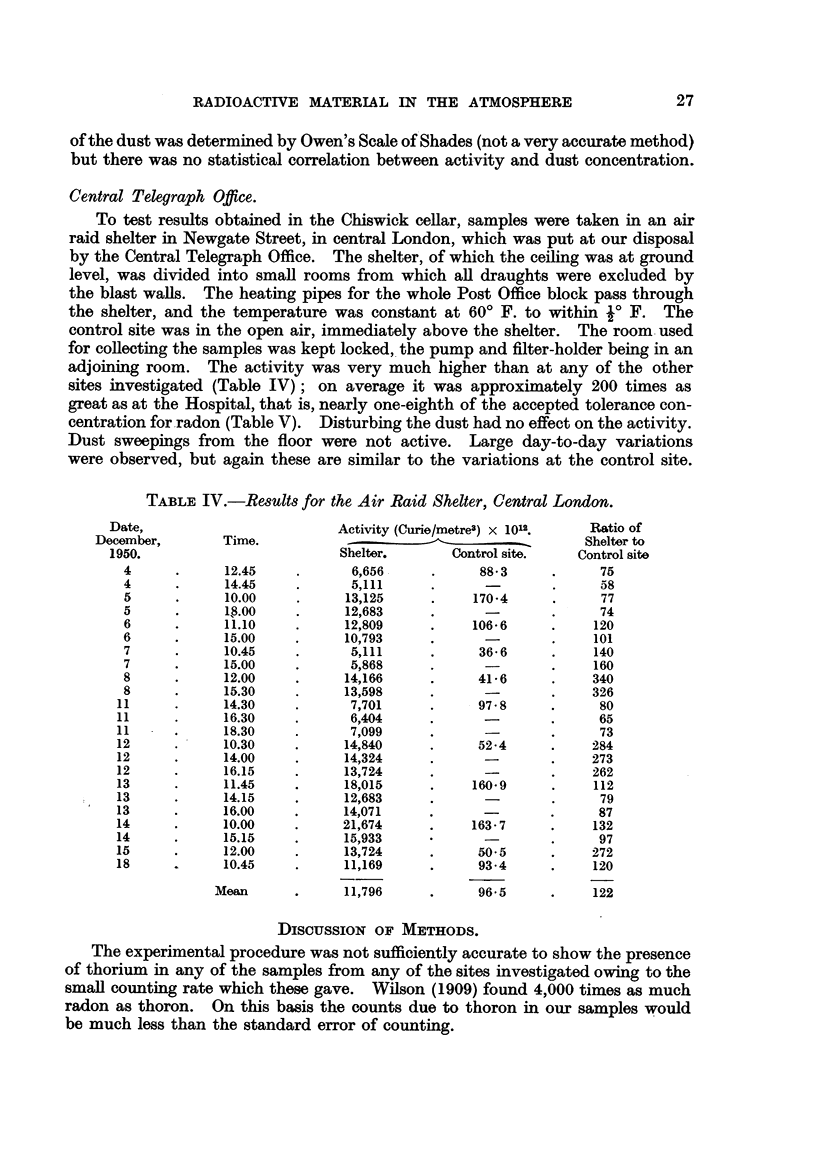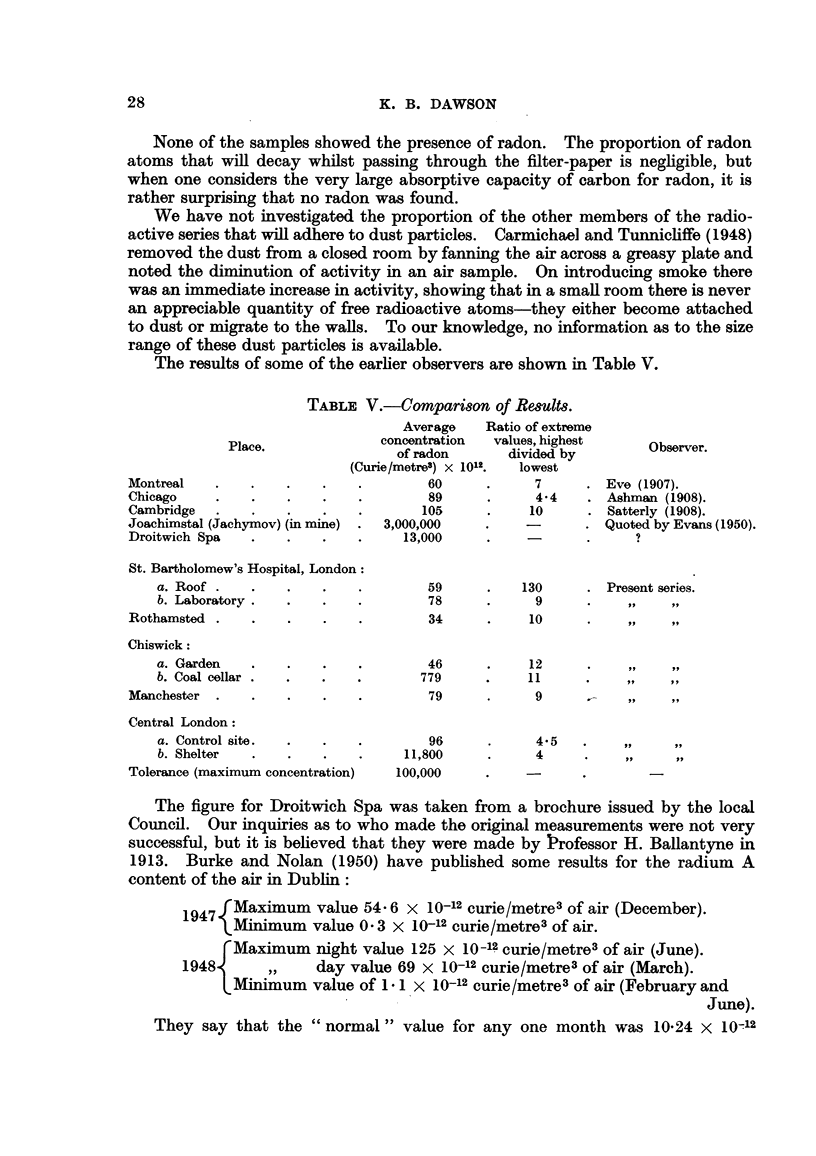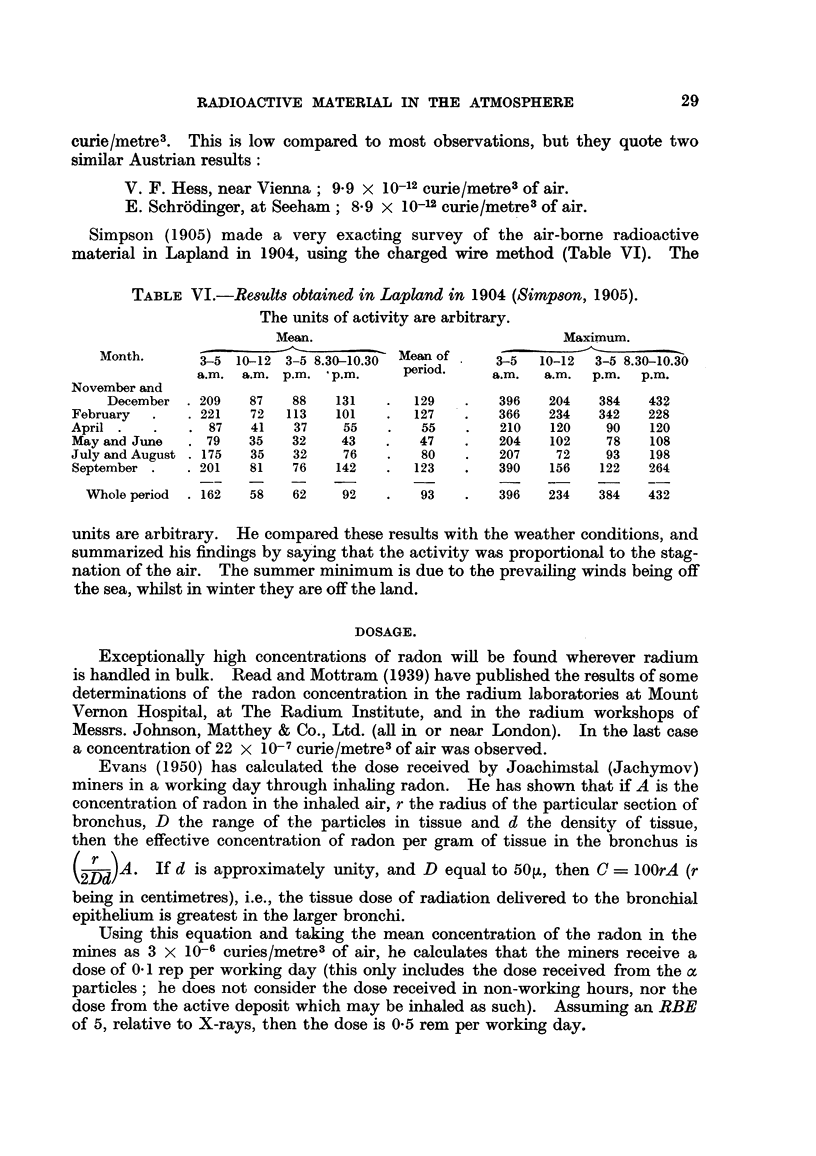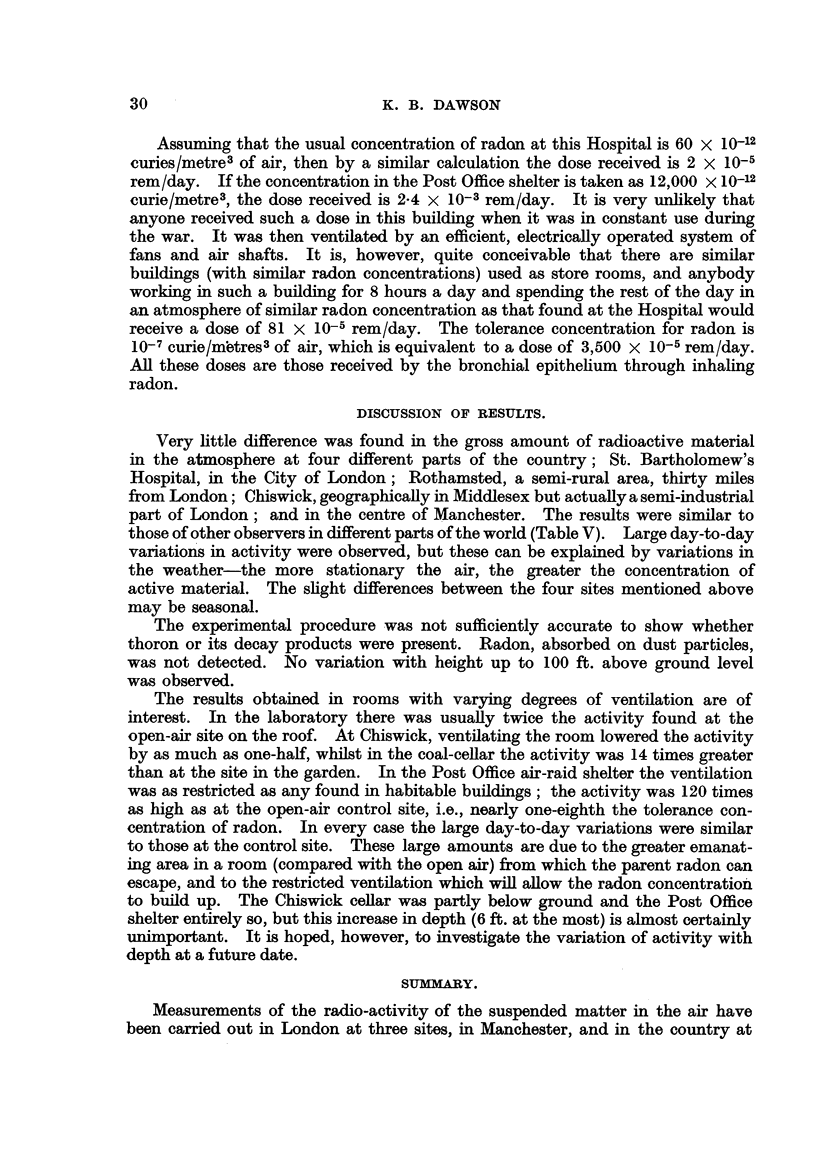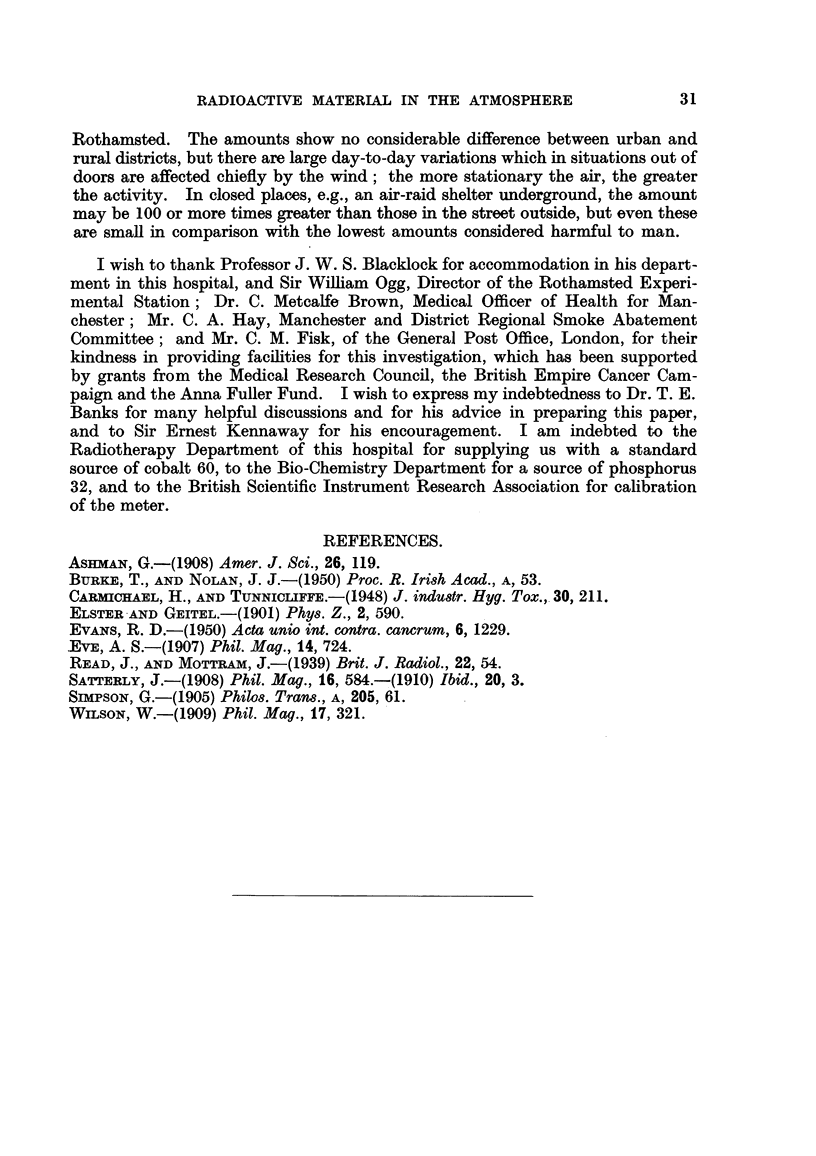# Radioactive Material in the Atmosphere

**DOI:** 10.1038/bjc.1952.3

**Published:** 1952-03

**Authors:** K. B. Dawson


### RADIOACTIVE MATERIAL IN THE ATMOSPHERE.

K. B. DAWSON.

*From the Pathological Department, St. Bartholomew's Hospital, London, E.C.*1.

Received for publication February 11, 1952.

RADIUM and thorium are widely distributed throughout the earth's crust, although, as yet, no large deposits have been found (it has been estimated that uranium and thorium exist in as much as 4 and 12 parts per million respectively). Both these materials decay through a radioactive series, and in each case one member of the series is gaseous. These two radioactive gases escape into the atmosphere where their respective decay products will be formed and these products will probably attach themselves to dust particles. There are other sources of naturally occurring radioactive material (actinium, which decays to gaseous actinon ; potassium ; rubidium and carbon 14), but these are insignificant compared to members of the radium and thorium series.

The presence of radioactive material in the atmosphere was first observed by Elster and Geitel (1901). Their observations were confirmed by other workers in various parts of the world. Most of these early determinations were made by the charged wire method, which gave quite good qualitative results. An improvement was made when A. S. Eve (1907), following a suggestion of Rutherford, absorbed radon on activated coconut charcoal, the apparatus being calibrated by radon from a measured source of radium bromide.

### METHOD.

The method used was to draw air at a constant rate through a filter-paper, and then to measure the deposited activity under an end-window Geiger counter. The filter-paper was Whatman No. 41, and the filter-holder had a 1-inch-diameter circular aperture. The rate of air flow depended on the length of tubing connecting the pump and filter-holder, but was always between 30 and 35 litres a minute. It was measured by a U-tube manometer, and controlled by a screw clip. The manometer was calibrated by a dry gas meter, the accuracy of which was determined for us by Mr. Redding of the British Scientific Instrument Research Association. The sample was placed at once in a brass tray under a General Electric Co. type EHM 2 Geiger counter, housed in a lead castle, and a decay-curve was taken over a period of half an hour. The collecting time was 20 minutes, which does not necessarily give the maximum measurable activity, but allows the experiment to be completed in an hour.

The efficiency of the filter was examined in a series of experiments in which one, two or three filter-papers of different types were used in series as a backing for the Whatman No. 41 filter-paper, and their activity measured. The Whatman No. 41 was found to be at least 99 per cent efficient. The activity of a uranium oxide source was measured before each sample, and the counting rate adjusted proportionally so that this standard source would give a constant figure. To make the results comparable, the data were adjusted to a pumping rate of 35 litres a minute. This method was suggested by Dr. T. E. Banks, and the apparatus was assembled by Mr. R. E. Waller of this laboratory.

The experimental difficulties in calibrating the apparatus with a known source of radon were prohibitive ; consequently, we decided to use a theoretical decay curve corrected for the efficiency of the Geiger counter in measuring the particles from individual members of the radium and thorium series. The geometrical efficiency of the counter is 15·4 per cent. As it was not possible to obtain standard samples of each member of the radium and thorium series, we determined the efficiency of the counter for measuring the $\beta$ particles from three radioactive isotopes. These efficiencies were plotted against energy, and the efficiency for the $\beta$-emitting members of the radium and thorium series were read from this graph.

The efficiency of the counter for measuring $\alpha$ particles cannot be determined in the same way as for $\beta$ particles, since an unknown number of $\alpha$ particles will be absorbed in the filter-paper. In order to calculate the $\alpha$ efficiency, a dust sample was taken and counted in the usual way ; a piece of aluminium foil (thick enough to absorb all the $\alpha$ particles) was then inserted between the filter-paper and the Geiger counter, and the count was continued. This experiment was repeated for a series of samples. The proportion of $\alpha$ to $\beta$ particles emitted by the dust was deduced theoretically, and the proportion of $\alpha$ particles which were counted was determined from the drop in counting rate caused by interposing the aluminium foil. The efficiency of the counter for measuring $\alpha$ particles was then deduced from these two figures together with the previously determined efficiency of the counter for $\beta$ particles. It was assumed that the proportion of $\alpha$ particles absorbed in the filter-paper was the same in every dust sample, and that the counter efficiency was the same for all $\alpha$ emitters. The counter used does not record $\gamma$ rays.

These counting efficiencies were then applied as corrections to the theoretical decay curve which can then be used in the quantitative estimation of the individual samples and in their analysis to determine the proportion of radium A and thorium A present.

### RESULTS.

Samples were taken at six different sites during 1950 (Table II) ; the individual sites are described below.

*Pathology Department at St. Bartholomew's Hospital.*

(a) *Roof.*—This site was chosen mainly on the grounds of convenience, but it is in a reasonably exposed position in the centre of the city ; it is 78 ft. above ground level, but a series of samples taken at various levels on the fire-escape attached to the building showed no significant variation with height. Dust samples were taken daily from December 1949, to June 1950. These samples showed large day-to-day variations in activity, and when more than one sample was taken in one day, there were often considerable differences. Table I shows the activity of the samples for the months of January and May, whilst Table II shows the monthly means.

The most obvious cause of these variations is the weather. The reports of the Meteorological Office give data for Kew Observatory and London Airport.

The data for Kew were taken unless there was a significant difference between these two sites, in which case that day was ignored or mean values were taken. Atmospheric pressure, temperature, humidity, wind force and direction and precipitation were considered. Activity varied directly with pressure and absolute

TABLE I.—*Results obtained on the Roof of the Pathological Department, St. Bartholomew's Hospital, during January and May,* 1950.

January.

| Day of month. | Mean activity (Curies/metre$^3$) $\times 10^{12}$. | Number of samples. |
|---|---|---|
| 2 | 80·5 | 1 |
| 3 | 56·2 | 1 |
| 4 | 41·6 | 1 |
| 5 | 39·8 | 1 |
| 6 | 26·5 | 3 |
| 7 | 58·0 | 1 |
| 9 | 381·8 | 2 |
| 10 | 148·9 | 1 |
| 11 | 30·6 | 2 |
| 12 | 53·9 | 1 |
| 13 | 85·5 | 1 |
| 16 | 66·9 | 1 |
| 17 | 42·9 | 2 |
| 18 | 67·5 | 1 |
| 19 | 67·8 | 1 |
| 20 | 55·8 | 2 |
| 23 | 45·1 | 1 |
| 24 | 56·8 | 1 |
| 25 | 59·8 | 1 |
| 26 | 76·3 | 2 |
| 27 | 243·6 | 2 |
| 30 | 239·5 | 3 |
| 31 | 137·6 | 2 |
| Mean | 94·1 | Total 34 |

May.

| Day of month. | Mean activity (Curies/metre$^3$) $\times 10^{12}$. | Number of samples. |
|---|---|---|
| 2 | 3·1 | 2 |
| 3 | 28·7 | 1 |
| 4 | 20·5 | 1 |
| 5 | 24·0 | 2 |
| 8 | 85·5 | 1 |
| 10 | 57·4 | 1 |
| 11 | 31·9 | 2 |
| 12 | 48·9 | 1 |
| 15 | 25·9 | 2 |
| 16 | 16·7 | 1 |
| 17 | 15·1 | 1 |
| 18 | 37·2 | 1 |
| 22 | 6·6 | 1 |
| 23 | 13·2 | 1 |
| 25 | 21·4 | 1 |
| 26 | 24·3 | 2 |
| 30 | 21·4 | 1 |
| Mean | 28·4 | Total 22 |

humidity, and inversely with wind force. Neither rainfall nor temperature had any effect. The figures for wind direction were not sufficiently precise to show any relation to activity, but Satterly (1910), after a fairly exhaustive inquiry, showed that the activity increased, the greater the path of the wind overland. Samples taken here on foggy days were very active, but no more active than on some other days without fog.

(b) *Laboratory.*—During February, March and April, of 1950 dust samples were taken in the laboratory on the fourth floor of the Pathological Department, immediately beneath the collecting site on the roof, which showed corresponding variations, but were generally twice as active, due no doubt to the greater emanating surface of the six walls (the parent radium is found in most materials and can often be detected in brick dust) and to the lack of air currents.

*Rothamsted Experimental Station, Hertfordshire.*

Through the kindness of the Director of the Rothamsted Experimental Station samples were taken during June and July at the meteorological enclosure, which is in an exposed site at the top of a small hill in a semi-rural area, about 30 miles from London. These showed a lower average, and much less variation than those at the Hospital; those taken in the afternoon were usually less active

TABLE II.—*Summary of Results at all Collecting Sites.*

The monthly mean is calculated from the mean daily samples.

| Site. | Month, 1949–50. | No. of days. | No. of samples. | Mean activity (Curies/metre$^3$) $\times 10^{12}$. | Standard deviation. | Highest activity. | Lowest activity. | Activity : highest divided by lowest. | Ratio : Laboratory to roof. |
|---|---|---|---|---|---|---|---|---|---|
| Pathological Department, St. Bartholomew's Hospital : | | | | | | | | | |
| (*a*) Roof | December | 5 | 6 | 121·7 | 56·9 | 227·2 | 43·8 | 5·2 | |
| | January | 23 | 34 | 94·1 | 83·8 | 384·9 | 22·4 | 17·2 | |
| | February | 18 | 33 | 34·6 | 21·2 | 121·8 | 10·1 | 12·1 | |
| | March | 12 | 15 | 51·3 | 45·5 | 184·9 | 10·4 | 17·8 | |
| | April | 4 | 4 | 42·3 | 24·6 | 79·5 | 11·0 | 7·2 | |
| | May | 17 | 22 | 28·4 | 19·6 | 85·5 | 2·9 | 29·1 | |
| | June | 5 | 8 | 63 | 21·2 | 113·6 | 24·1 | 4·7 | |
| | Whole period | 84 | 122 | 59·2 | 59·6 | 384·9 | 2·9 | 131·2 | |
| (*b*) Laboratory | February | 18 | 20 | 76·6 | 28 | 116·7 | 40·1 | 2·9 | 2·4 |
| | March | 16 | 19 | 94 | 55·6 | 229 | 25·7 | 8·9 | 1·9 |
| | April | 3 | 3 | 106·1 | 33·2 | — | — | — | 2·0 |
| | May | 9 | 10 | 41·4 | 11·5 | 60·9 | 26·8 | 2·3 | 1·6 |
| | Whole period | 46 | 52 | 77·7 | 43·4 | 229 | 25·7 | 8·9 | 2·0 |
| Rothamsted | July | 26 | 52 | 34·4 | 2·5 | 78·9 | 7·6 | 10·4 | |
| Chiswick : | | | | | | | | | |
| (*a*) Control site | August | 20 | 34 | 45·7 | 39·5 | 197·8 | 12·5 | 16·3 | |
| (*b*) Cellar | ,, | 13 | 34 | 779 | 456 | 2,282 | 213 | 11·0 | |
| Manchester | November | 14 | 43 | 78·9 | 32·7 | 160·9 | 18·3 | 8·8 | |
| Central London : | | | | | | | | | |
| (*a*) Control site | December | 11 | 11 | 96·5 | 17·7 | 170·4 | 36·6 | 4·7 | |
| (*b*) Air raid shelter | ,, | 11 | 23 | 11,780 | 3,870 | 21,675 | 5,110 | 4·2 | |

than those taken in the morning. Rothamsted is a third-order station of the Meteorological Office and more accurate correlation of weather phenomena and activity were possible; the conclusions drawn from the Hospital results were confirmed. The activity was greater if the ground was dry or drying. A 100-ft. high wooden tower was available, from which samples were taken at 10 ft. intervals. Again, there was no significant variation with height.

*Chiswick.*

During August and September results were obtained in the garden of a private house beside the River Thames in Chiswick (West London), which were intermediate between those of Rothamsted and the Hospital. In a closed room ventilation by opening all possible doors and windows (about one-twelfth total wall and floor space) lowered the activity by as much as one-half. A series of samples were taken in the coal cellar beneath the house. The floor of the cellar was only 4 ft. below street level; there was some ventilation from a window and the door leading to the main part of the house. The cellar had not been used for several weeks. In order to avoid disturbing the dust, the pump and filter-holder were kept outside the cellar (Table III). On an average the cellar showed 14 times as much activity as the control site in the garden. Some samples taken in the house showed that the large activity was confined to the cellar. Samples taken after disturbance of the dust in the Chiswick cellar showed no appreciable increase; the variations were similar to those at the control site, and must therefore have been caused by the weather. The coal dust which covered the floor and walls was not active.

TABLE III.—*Comparison of the Cellar and Exposed Control Site at Chiswick.*

| Day of the month, August, 1950. | Mean daily activity (Curies/metre$^3$ of air) $\times 10^{12}$. | |
|---|---|---|
| | Cellar. | Control site. |
| 14 | 685 | 40·1 |
| 16 | 681 | 18·6 |
| 18 | 435 | 14·8 |
| 21 | 549 | 59·7 |
| 22 | 912 | 170·7 |
| 23 | 1,305 | 65·0 |
| 24 | 243 | 22·7 |
| 25 | 228 | 17·3 |
| 28 | 745 | 58·0 |
| 29 | 461 | 19·2 |
| 30 | 688 | 85·1 |
| 31 | 1,719 | 98·4 |
| 1 | 1,483 | 36·3 |
| Mean | 779 | 54·3 |

*Manchester.*

The collecting site was a window-ledge 8 ft. above street level on a building belonging to the Medical Officer of Health's Department, in the city centre. Samples taken during November, 1950, were, on average, the highest yet found, except for the samples taken at this Hospital in December and January (Table II). In Manchester atmospheric pollution is greatest in November. The mass of the dust was determined by Owen's Scale of Shades (not a very accurate method) but there was no statistical correlation between activity and dust concentration.

*Central Telegraph Office.*

To test results obtained in the Chiswick cellar, samples were taken in an air raid shelter in Newgate Street, in central London, which was put at our disposal by the Central Telegraph Office. The shelter, of which the ceiling was at ground level, was divided into small rooms from which all draughts were excluded by the blast walls. The heating pipes for the whole Post Office block pass through the shelter, and the temperature was constant at 60° F. to within ½° F. The control site was in the open air, immediately above the shelter. The room used for collecting the samples was kept locked, the pump and filter-holder being in an adjoining room. The activity was very much higher than at any of the other sites investigated (Table IV); on average it was approximately 200 times as great as at the Hospital, that is, nearly one-eighth of the accepted tolerance concentration for radon (Table V). Disturbing the dust had no effect on the activity. Dust sweepings from the floor were not active. Large day-to-day variations were observed, but again these are similar to the variations at the control site.

TABLE IV.—*Results for the Air Raid Shelter, Central London.*

| Date, December, 1950. | Time. | Activity (Curie/metre³) × 10¹². Shelter. | Control site. | Ratio of Shelter to Control site |
|---|---|---|---|---|
| 4 | 12.45 | 6,656 | 88·3 | 75 |
| 4 | 14.45 | 5,111 | — | 58 |
| 5 | 10.00 | 13,125 | 170·4 | 77 |
| 5 | 18.00 | 12,683 | — | 74 |
| 6 | 11.10 | 12,809 | 106·6 | 120 |
| 6 | 15.00 | 10,793 | — | 101 |
| 7 | 10.45 | 5,111 | 36·6 | 140 |
| 7 | 15.00 | 5,868 | — | 160 |
| 8 | 12.00 | 14,166 | 41·6 | 340 |
| 8 | 15.30 | 13,598 | — | 326 |
| 11 | 14.30 | 7,701 | 97·8 | 80 |
| 11 | 16.30 | 6,404 | — | 65 |
| 11 | 18.30 | 7,099 | — | 73 |
| 12 | 10.30 | 14,840 | 52·4 | 284 |
| 12 | 14.00 | 14,324 | — | 273 |
| 12 | 16.15 | 13,724 | — | 262 |
| 13 | 11.45 | 18,015 | 160·9 | 112 |
| 13 | 14.15 | 12,683 | — | 79 |
| 13 | 16.00 | 14,071 | — | 87 |
| 14 | 10.00 | 21,674 | 163·7 | 132 |
| 14 | 15.15 | 15,933 | — | 97 |
| 15 | 12.00 | 13,724 | 50·5 | 272 |
| 18 | 10.45 | 11,169 | 93·4 | 120 |
| | Mean | 11,796 | 96·5 | 122 |

### DISCUSSION OF METHODS.

The experimental procedure was not sufficiently accurate to show the presence of thorium in any of the samples from any of the sites investigated owing to the small counting rate which these gave. Wilson (1909) found 4,000 times as much radon as thoron. On this basis the counts due to thoron in our samples would be much less than the standard error of counting.

None of the samples showed the presence of radon. The proportion of radon atoms that will decay whilst passing through the filter-paper is negligible, but when one considers the very large absorptive capacity of carbon for radon, it is rather surprising that no radon was found.

We have not investigated the proportion of the other members of the radioactive series that will adhere to dust particles. Carmichael and Tunnicliffe (1948) removed the dust from a closed room by fanning the air across a greasy plate and noted the diminution of activity in an air sample. On introducing smoke there was an immediate increase in activity, showing that in a small room there is never an appreciable quantity of free radioactive atoms—they either become attached to dust or migrate to the walls. To our knowledge, no information as to the size range of these dust particles is available.

The results of some of the earlier observers are shown in Table V.

TABLE V.—*Comparison of Results.*

| Place. | Average concentration of radon (Curie/metre³) × $10^{12}$. | Ratio of extreme values, highest divided by lowest | Observer. |
|---|---|---|---|
| Montreal | 60 | 7 | Eve (1907). |
| Chicago | 89 | 4·4 | Ashman (1908). |
| Cambridge | 105 | 10 | Satterly (1908). |
| Joachimstal (Jachymov) (in mine) | 3,000,000 | — | Quoted by Evans (1950). |
| Droitwich Spa | 13,000 | — | ? |
| St. Bartholomew's Hospital, London : | | | |
| *a.* Roof | 59 | 130 | Present series. |
| *b.* Laboratory | 78 | 9 | ,, ,, |
| Rothamsted | 34 | 10 | ,, ,, |
| Chiswick : | | | |
| *a.* Garden | 46 | 12 | ,, ,, |
| *b.* Coal cellar | 779 | 11 | ,, ,, |
| Manchester | 79 | 9 | ,, ,, |
| Central London : | | | |
| *a.* Control site | 96 | 4·5 | ,, ,, |
| *b.* Shelter | 11,800 | 4 | ,, ,, |
| Tolerance (maximum concentration) | 100,000 | — | — |

The figure for Droitwich Spa was taken from a brochure issued by the local Council. Our inquiries as to who made the original measurements were not very successful, but it is believed that they were made by Professor H. Ballantyne in 1913. Burke and Nolan (1950) have published some results for the radium A content of the air in Dublin :

1947 { Maximum value $54 \cdot 6 \times 10^{-12}$ curie/metre³ of air (December).

Minimum value $0 \cdot 3 \times 10^{-12}$ curie/metre³ of air.

1948 { Maximum night value $125 \times 10^{-12}$ curie/metre³ of air (June).

,, day value $69 \times 10^{-12}$ curie/metre³ of air (March).

Minimum value of $1 \cdot 1 \times 10^{-12}$ curie/metre³ of air (February and June).

They say that the "normal" value for any one month was $10{\cdot}24 \times 10^{-12}$

curie/metre$^3$. This is low compared to most observations, but they quote two similar Austrian results :

V. F. Hess, near Vienna ; $9{\cdot}9 \times 10^{-12}$ curie/metre$^3$ of air.
E. Schrödinger, at Seeham ; $8{\cdot}9 \times 10^{-12}$ curie/metre$^3$ of air.

Simpson (1905) made a very exacting survey of the air-borne radioactive material in Lapland in 1904, using the charged wire method (Table VI). The

TABLE VI.—*Results obtained in Lapland in* 1904 (*Simpson*, 1905).
The units of activity are arbitrary.

| Month. | Mean. | | | | Mean of period. | Maximum. | | | |
|---|---|---|---|---|---|---|---|---|---|
| | 3–5 a.m. | 10–12 a.m. | 3–5 p.m. | 8.30–10.30 p.m. | | 3–5 a.m. | 10–12 a.m. | 3–5 p.m. | 8.30–10.30 p.m. |
| November and December | 209 | 87 | 88 | 131 | 129 | 396 | 204 | 384 | 432 |
| February | 221 | 72 | 113 | 101 | 127 | 366 | 234 | 342 | 228 |
| April | 87 | 41 | 37 | 55 | 55 | 210 | 120 | 90 | 120 |
| May and June | 79 | 35 | 32 | 43 | 47 | 204 | 102 | 78 | 108 |
| July and August | 175 | 35 | 32 | 76 | 80 | 207 | 72 | 93 | 198 |
| September | 201 | 81 | 76 | 142 | 123 | 390 | 156 | 122 | 264 |
| Whole period | 162 | 58 | 62 | 92 | 93 | 396 | 234 | 384 | 432 |

units are arbitrary. He compared these results with the weather conditions, and summarized his findings by saying that the activity was proportional to the stagnation of the air. The summer minimum is due to the prevailing winds being off the sea, whilst in winter they are off the land.

### DOSAGE.

Exceptionally high concentrations of radon will be found wherever radium is handled in bulk. Read and Mottram (1939) have published the results of some determinations of the radon concentration in the radium laboratories at Mount Vernon Hospital, at The Radium Institute, and in the radium workshops of Messrs. Johnson, Matthey & Co., Ltd. (all in or near London). In the last case a concentration of $22 \times 10^{-7}$ curie/metre$^3$ of air was observed.

Evans (1950) has calculated the dose received by Joachimstal (Jachymov) miners in a working day through inhaling radon. He has shown that if $A$ is the concentration of radon in the inhaled air, $r$ the radius of the particular section of bronchus, $D$ the range of the particles in tissue and $d$ the density of tissue, then the effective concentration of radon per gram of tissue in the bronchus is $\left(\frac{r}{2Dd}\right)A$. If $d$ is approximately unity, and $D$ equal to $50\mu$, then $C = 100rA$ ($r$ being in centimetres), i.e., the tissue dose of radiation delivered to the bronchial epithelium is greatest in the larger bronchi.

Using this equation and taking the mean concentration of the radon in the mines as $3 \times 10^{-6}$ curies/metre$^3$ of air, he calculates that the miners receive a dose of $0{\cdot}1$ rep per working day (this only includes the dose received from the $\alpha$ particles ; he does not consider the dose received in non-working hours, nor the dose from the active deposit which may be inhaled as such). Assuming an *RBE* of 5, relative to X-rays, then the dose is $0{\cdot}5$ rem per working day.

Assuming that the usual concentration of radon at this Hospital is $60 \times 10^{-12}$ curies/metre$^3$ of air, then by a similar calculation the dose received is $2 \times 10^{-5}$ rem/day. If the concentration in the Post Office shelter is taken as $12{,}000 \times 10^{-12}$ curie/metre$^3$, the dose received is $2{\cdot}4 \times 10^{-3}$ rem/day. It is very unlikely that anyone received such a dose in this building when it was in constant use during the war. It was then ventilated by an efficient, electrically operated system of fans and air shafts. It is, however, quite conceivable that there are similar buildings (with similar radon concentrations) used as store rooms, and anybody working in such a building for 8 hours a day and spending the rest of the day in an atmosphere of similar radon concentration as that found at the Hospital would receive a dose of $81 \times 10^{-5}$ rem/day. The tolerance concentration for radon is $10^{-7}$ curie/mètres$^3$ of air, which is equivalent to a dose of $3{,}500 \times 10^{-5}$ rem/day. All these doses are those received by the bronchial epithelium through inhaling radon.

### DISCUSSION OF RESULTS.

Very little difference was found in the gross amount of radioactive material in the atmosphere at four different parts of the country; St. Bartholomew's Hospital, in the City of London; Rothamsted, a semi-rural area, thirty miles from London; Chiswick, geographically in Middlesex but actually a semi-industrial part of London; and in the centre of Manchester. The results were similar to those of other observers in different parts of the world (Table V). Large day-to-day variations in activity were observed, but these can be explained by variations in the weather—the more stationary the air, the greater the concentration of active material. The slight differences between the four sites mentioned above may be seasonal.

The experimental procedure was not sufficiently accurate to show whether thoron or its decay products were present. Radon, absorbed on dust particles, was not detected. No variation with height up to 100 ft. above ground level was observed.

The results obtained in rooms with varying degrees of ventilation are of interest. In the laboratory there was usually twice the activity found at the open-air site on the roof. At Chiswick, ventilating the room lowered the activity by as much as one-half, whilst in the coal-cellar the activity was 14 times greater than at the site in the garden. In the Post Office air-raid shelter the ventilation was as restricted as any found in habitable buildings; the activity was 120 times as high as at the open-air control site, i.e., nearly one-eighth the tolerance concentration of radon. In every case the large day-to-day variations were similar to those at the control site. These large amounts are due to the greater emanating area in a room (compared with the open air) from which the parent radon can escape, and to the restricted ventilation which will allow the radon concentration to build up. The Chiswick cellar was partly below ground and the Post Office shelter entirely so, but this increase in depth (6 ft. at the most) is almost certainly unimportant. It is hoped, however, to investigate the variation of activity with depth at a future date.

### SUMMARY.

Measurements of the radio-activity of the suspended matter in the air have been carried out in London at three sites, in Manchester, and in the country at

Rothamsted. The amounts show no considerable difference between urban and rural districts, but there are large day-to-day variations which in situations out of doors are affected chiefly by the wind ; the more stationary the air, the greater the activity. In closed places, e.g., an air-raid shelter underground, the amount may be 100 or more times greater than those in the street outside, but even these are small in comparison with the lowest amounts considered harmful to man.

I wish to thank Professor J. W. S. Blacklock for accommodation in his department in this hospital, and Sir William Ogg, Director of the Rothamsted Experimental Station ; Dr. C. Metcalfe Brown, Medical Officer of Health for Manchester ; Mr. C. A. Hay, Manchester and District Regional Smoke Abatement Committee ; and Mr. C. M. Fisk, of the General Post Office, London, for their kindness in providing facilities for this investigation, which has been supported by grants from the Medical Research Council, the British Empire Cancer Campaign and the Anna Fuller Fund. I wish to express my indebtedness to Dr. T. E. Banks for many helpful discussions and for his advice in preparing this paper, and to Sir Ernest Kennaway for his encouragement. I am indebted to the Radiotherapy Department of this hospital for supplying us with a standard source of cobalt 60, to the Bio-Chemistry Department for a source of phosphorus 32, and to the British Scientific Instrument Research Association for calibration of the meter.

### REFERENCES.

ASHMAN, G.—(1908) *Amer. J. Sci.*, **26**, 119.
BURKE, T., AND NOLAN, J. J.—(1950) *Proc. R. Irish Acad.*, A, 53.
CARMICHAEL, H., AND TUNNICLIFFE.—(1948) *J. industr. Hyg. Tox.*, **30**, 211.
ELSTER AND GEITEL.—(1901) *Phys. Z.*, **2**, 590.
EVANS, R. D.—(1950) *Acta unio int. contra. cancrum*, **6**, 1229.
EVE, A. S.—(1907) *Phil. Mag.*, **14**, 724.
READ, J., AND MOTTRAM, J.—(1939) *Brit. J. Radiol.*, **22**, 54.
SATTERLY, J.—(1908) *Phil. Mag.*, **16**, 584.—(1910) *Ibid.*, **20**, 3.
SIMPSON, G.—(1905) *Philos. Trans.*, A, **205**, 61.
WILSON, W.—(1909) *Phil. Mag.*, **17**, 321.